# Predictive Value of Coronary Calcifications for Future Cardiac Events in Asymptomatic Patients with Severe Chronic Kidney Disease

**DOI:** 10.31083/j.rcm2511398

**Published:** 2024-11-08

**Authors:** Martin Greif, Korbinian Lackermair, Matthias Wessely, Franz von Ziegler, Alexander Becker

**Affiliations:** ^1^Department of Cardiology, Ludwig-Maximilians-University, 80539 Munich, Germany

**Keywords:** coronary calcification, chronic kidney disease, cardiovascular risk, risk reduction, predictive value

## Abstract

**Background:**

Coronary calcification is a well-established risk factor for cardiovascular events. This retrospective study sought to determine the predictive value of coronary calcification in a specific group of patients with chronic kidney disease.

**Methods:**

We included 1094 asymptomatic patients (724 males, 370 females, age 62 ± 9.3 years) referred for cardiological examination. Patents were divided into two groups depending on their renal function. Coronary calcification was determined with a multi-slice computer tomography (CT) scanner. For quantification of coronary calcification the Agatston score was calculated. Over a mean follow up period of 6.2 ± 1.3 years we observed the rate of cardiovascular events (185 events, 61 myocardial infarctions, 103 revascularizations, 21 cardiac deaths).

**Results:**

The calcium score was significantly higher in patients with severe kidney disease (glomerular filtration rate (GFR) ≤30 mL/min/1.72 m^2^) compared with those with normal to moderate reduced renal function (GFR ≥30 mL/min/1.72 m^2^) (207 ± 190 *vs*.121 ± 169, *p* ≤ 0.001). The event rate in patients with severe impaired renal function was significantly higher compared to patients with normal to moderate reduced renal function (20.6% *vs*. 14.8%, *p* = 0.0001). The hazard ratio for cardiovascular events increased constantly with the calcium score in both groups. The hazard ratio in patients with severe kidney disease was significantly lower compared to patients in corresponding groups with regular to moderate reduced renal function (7.3 *vs*. 9.3, *p* = 0.01). No cardiac events were observed in patients with a calcium score of 0.

**Conclusions:**

We could demonstrate that risk prediction with the calcium score is possible in patients with severe chronic kidney disease even if the calcium score overestimates the risk for future cardiovascular events compared to patients with normal to moderate reduced renal function.

## 1. Introduction

Calcification of the coronary artery is pathognomonic of coronary atherosclerosis and has been confirmed by intravascular ultrasound study and histopathology [1].

Therefore, coronary calcification is a well-established marker for coronary artery disease and predictor for cardiovascular morbidity and cardiovascular death [2]. Several studies demonstrated an association between future cardiovascular events and the amount of coronary calcium (CAC) [3, 4, 5, 6]. CAC screening can therefore be used for risk stratification for future cardiovascular events [4, 5]. Quantification of CAC can be done noninvasively by cardiac computed tomography (CT) [2, 4, 5]. The individual amount of CAC provides an individual risk prediction that is superior to conventional risk factors scores like the ATP III Score or PROCAM score [5]. A CAC of zero, is associated with a high negative predictive value [6]. In, higher scores, e.g., above the 75th percentile or above 400 are associated with an elevated cardiovascular risk independent of underlying risk factors [4].

Coronary calcifications can reflect the individual extent of coronary atherosclerosis. Thus, extensive calcification can be associated with coronary stenosis. The specificity of CAC in the diagnosis of relevant coronary stenosis is limited. The absence of coronary calcification, a score of zero, has a high negative predictive value and can be used for the exclusion of coronary artery disease.

Patients with severe chronic kidney disease (CKD) are at high risk for cardiovascular disease independent from concomitant risk factors [7, 8]. In addition, classic cardiovascular risk factors such as hypertension are common in patients with severe CKD. In addition to classic atherosclerotic lesions which are located in the arterial intima and are the consequences of multifocal, smoldering, immunoinflammatory processes [9] in patients with severe CKD media sclerosis is frequent. In media sclerosis, also known as Monckeberg’s sclerosis, muscular arteries undergo non-inflammatory medial calcification [10], which is asymptomatic. Progression of media sclerosis results in the loss of the cushioning function in blood vessels, thus inducing pseudo hypertension, left ventricular hypertrophy and altered coronary perfusion [10, 11, 12].

Because of this additional mechanism of vascular calcification in severe CKD patients, the predictive value of CAC could be altered. Therefore we investigated the predictive value of CAC in a population of severe CKD patients and compared it to a population with regular to moderate reduced renal function.

## 2. Methods

### 2.1 Study Protocol

**Fig. 1.**
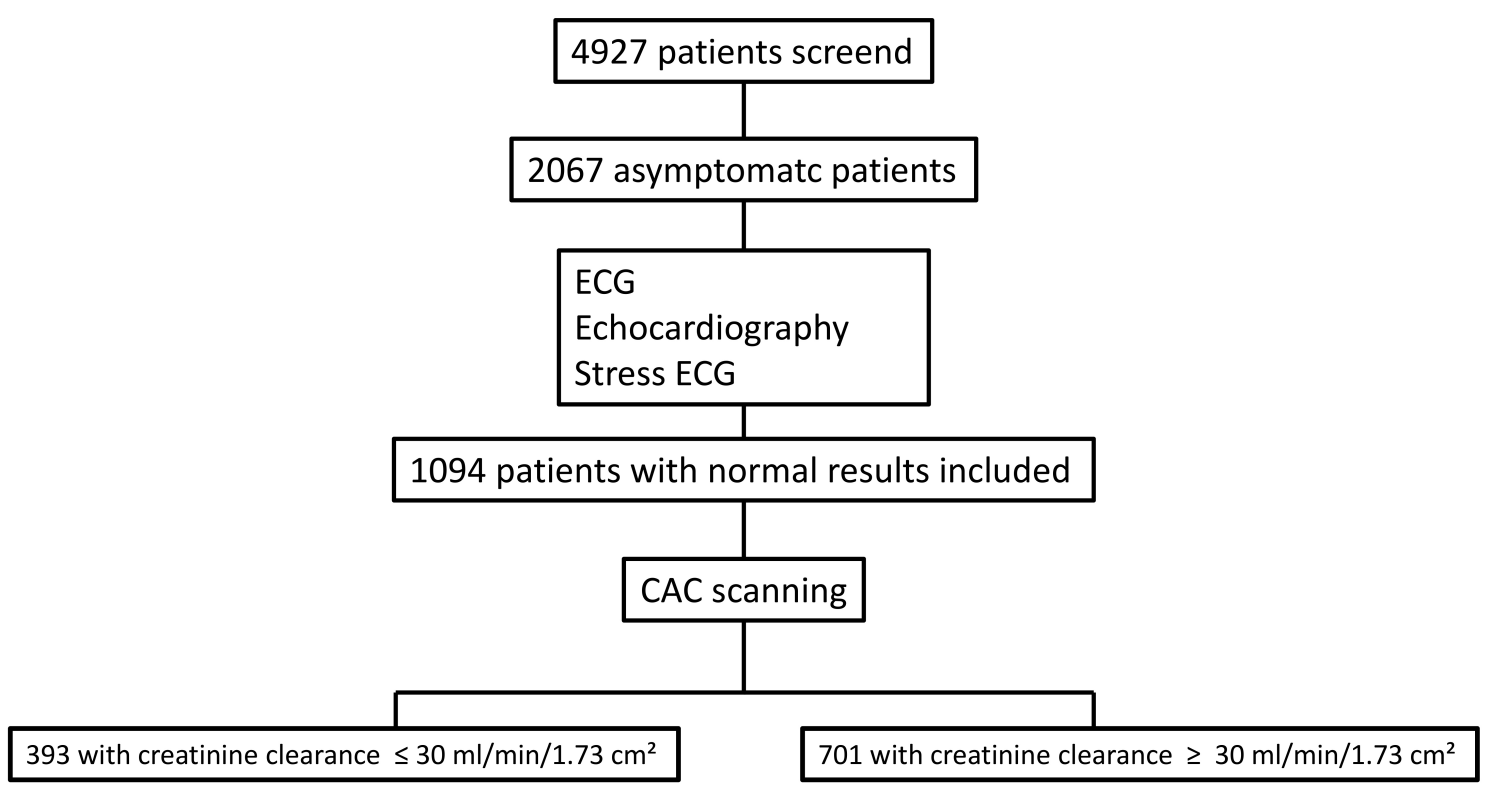
**Study flowchart**. ECG, electrocardiogram; CAC, coronary artery calcium.

The research protocol was authorized by the local Clinical Institutional Review Board and is in accordance with the declaration of Helsinki. We retrospectively examined 1094 consecutive patients sent for a preventive cardiology examination between 2000 and 2005. All patients were asymptomatic and underwent clinical examination, echocardiogram (ECG), stress ECG and echocardiography. Patients were enrolled after they gave written consent to undergo multi-slice CT in order to assess the CAC, and a follow-up interview (Fig. 1).

### 2.2 Risk Factors

In all participants we evaluated common cardiovascular risk factors by personal interview and the medical history. Arterial blood pressure, high-density lipoprotein (HDL) cholesterol level, low-density lipoprotein (LDL) cholesterol level, triglyceride level, blood glucose level and creatinine clearance were determined. In patients with statin therapy, hyperlipidemia was assumed, as no patients with known cardiovascular disease were included. Smokers were predefined as active smokers at the start of the study. According to the creatinine clearance, patients were divided into two groups. Patients with a creatinine clearance ≤30 mL/min/1.73 cm^2^ in group 1 and patients with a creatinine clearance ≥30 mL/min/1.73 cm^2^ into group 2.

### 2.3 Coronary Artery Calcium Scanning

CAC scanning was done using a Siemens multi-slice CT scanner (Somatron Sensation 4 or 16, Siemens Medical Solutions, Forchheim, Germany) in the high-resolution mode. ECG-triggered images of 100 ms duration were acquired at 80% of the R-R interval during one end-inspiratory breath-holding period. A total of 40 three mm-thick slices were obtained capping the whole heart. Coronary calcifications were automatically defined as lesions with a density >130 Hounsfield units (HU) in more than 3 continuous pixels. To quantify coronary calcium, the Agatston score was used, which constitutes the product of the lesion’s surface area and a weighting factor ranging from 1 to 4, which was assigned according to the peak density of the lesion [13].

### 2.4 Clinical Follow-Up

Patients were contacted after a mean observation time of 6.2 ± 1.3 years. Cardiovascular events were evaluated by a standardized telephone interview. In cases of hospital admission or further cardiologic examinations the patient’s medical records were verified for symptoms of chest discomfort, dyspnea, myocardial infarction (MI), and coronary revascularization. The treating physicians were not aware of the CAC score of the patients.

### 2.5 Clinical Endpoints

The primary endpoint was a combined endpoint of cardiac events including cardiac death, myocardial infarction, and revascularization. MI was defined using the World Health Organization (WHO)-Monica definition as the presence of at least 2 of the following: ongoing chest pain on hospital admission, typical ECG changes, elevation of serum creatine kinase levels up to twice the upper limit with an elevated creatine kinase-mass concentration (MB) fraction or troponin level without prior coronary intervention. Death due to coronary artery disease was assumed if the death was found to be due to coronary atherosclerosis by autopsy, occurred within 1 hour after onset of prolonged severe chest pain, or occurred during hospital admission because of an MI. Cardiac death was confirmed by autopsy in 27%, in 66% prior MI was proven by ECG and laboratory testing. In 7% clinical symptoms indicated a fatal infarction.

Revascularization (percutaneous coronary intervention or coronary artery bypass graft) was reviewed by standardized telephone interviews and the patients’ medical records. Coronary interventions had to be verified by reports from the performing physician.

### 2.6 Statistical Analysis

Statistical analyses were done using the SPSS software package (version 19.0, SPSS Inc. Chicago, IL, USA). Calcium score (CS) was expressed as mean score ± standard deviation except where indicated. Because of the non-normality, median, 1st and 3rd quartile were also given, and statistical analysis was performed on the base 10 log of the transformed Agatston score + 1. To compare score values in different risk groups we used the Wilcoxon signed rank test for unpaired data. We performed a two tailed test. A *p*-value under 0.05 was considered to indicate statistical significance.

We used logistic regression analysis in a univariate and multivariate model to calculate the risk ratio estimates and 95% confidence intervals for cardiac death, MI and revascularization in dependence of different score groups (patients with a score of 0 served as the reference group) and to calculate the risk ratio of cardiovascular risk factors (patients without cardiovascular risk factors served as the reference group) for calcium scores above 400.

We calculated the odds ratio for a calcium score above 400 in dependence of different risk factors including hypertension, hypercholesterolemia and diabetes, sex and age. After a univariate analysis we established a multivariable Cox proportional hazards model. To verify the assumption of proportional hazards we performed an analysis of the calculated risk ratios as described by Hosmer and Lemeshow. To account for the inflation of the type I error due to multiple testing we performed the Bonferroni adjustment. The significance level was set at 0.05/4 = 0.0125.

## 3. Results

1094 individuals (724 males and 370 females, aged 62 ± 9.3 years) were included in the study. 393 individuals had a creatinine clearance ≤30 mL/min/1.73 cm^2^ (group 1) and 701 patients had a creatinine clearance ≥30 mL/min/1.73 cm^2^ (group 2). The mean observation time was 6.2 ± 1.3 years. Patient characteristics for both groups are listed in Table 1. The mean number of risk factors per person in each group was 1.9 ± 1.0. There was no significant difference in age and risk factor distribution between the two study groups.

**Table 1.** **Baseline characteristics of 1094 patient included in the study**.

		All patients		Severe CKD		Normal to moderately reduced renal function		*p*-value
		n	%	n	%	n	%	
Patients		1094		393		701		
Male		724	66.2	270	68.7	454	64.8	0.25
Female		370	33.8	123	31.3	247	35.2	0.3
Age (yrs.)		62.0 ± 9.3		63.0 ± 10.4		61.4 ± 11.2		0.28
BMI (kg/m^2^)		26.8 ± 5.4		28.0 ± 5.7		26.1 ± 5.5		0.19
Arterial hypertension		587	53.6	205	52.2	382	54.5	0.27
Hyperlipidemia		629	57.5	221	56.2	408	58.2	0.19
	Statin therapy	249	22.8	118	30	131	18.7	0.004
Diabetes		184	16.8	64	16.3	120	17.1	0.19
Family history of CAD		389	35.6	141	35.9	248	35.4	0.30
Smoking		313	28.6	116	29.5	197	28.1	0.24
	Mean number of risk factors	1.9		1.9		1.9	1.9	0.35
CAC-Score (mean ± SD)		152 ± 127		207 ± 190		121 ± 169		<0.001
	Median	87		157		43		
	1st	29		42		13		
	3rd	208		315		227		

CKD, chronic kidney disease; BMI, body mass index; CAD, coronary artery disease; CAC, coronary artery calcification; yrs, years.

The mean CAC score was 152 ± 127 in all patients. The average calcium score was significantly higher in patients with severe kidney dysfunction than in patients with normal to moderate reduced renal function (207 ± 190 *vs*. 121 ± 169, *p* < 0.001). Coronary calcifications could be excluded in 180 individuals (44 in group 1 and 136 in group 2).

The mean score in females was significantly lower compared to males (101 ± 78 *vs*. 178 ± 141, *p* < 0.01). We found this difference in patients with normal to moderate reduced renal function (88.6 ± 71.2 *vs*. 138 ± 102, *p* < 0.01) and in patients with severe CKD (174 ± 153 *vs*. 222 ± 171, *p* < 0.01).

185 cardiovascular events (MI, myocardial revascularization, cardiac death) were observed. There was a significantly higher event rate of all cardiovascular events in patients with severe reduced renal function, *p* = 0.001. 19 patients in group 1 and 21 patients in group 2 died from cardiac death (*p* = 0.03). 107 patients suffered from MI, 46 in group 1 and 61 in group 2. 72 patients in group 1 and 94 patients in group 2 underwent myocardial revascularization, *p* < 0.01. The distribution of cardiovascular events and event rates are given in Table 2.

**Table 2.** **Event rates of all study patients and patients in severe CKD and normal to moderate reduced renal function**.

	All patients		Severe CKD		Normal to moderately reduced renal function		*p*-value
	n	%	n	%	n	%	
CV events	185	16.9	81	20.6	104	14.8	0.0001
Myocardial infarction	61	5.6	28	7.1	33	4.7	0.01
Cardiac death	21	1.9	11	2.8	10	2.4	0.04
Revascularisation	103	9.4	42	10.7	61	8.7	0.01
Angiography	201	18.4	79	20.1	122	17.4	0.03

CKD, chronic kidney disease; CV, cardiovascular.

In Tables 3,4, the risk ratio of patients for a calcium score above 400 is given in dependence of different cardiovascular risk factors. In a multivariate analysis, we could identify a significant correlation between a calcium score above 400 and age, male sex, hyperlipidemia, diabetes, smoking, and hypertension. In addition, severe renal insufficiency could be identified as an independent risk factor for an elevated calcium score.

**Table 3.** **Univariate analysis, hazard ratio of a calcium score above 400 for different risk factors**.

Univariate	Hazard ratio	*p*-value
Age	2.3	1.0–4.1
Male sex	2.6	1.5–4.4
Hyperlipidämia	9.7	3.7–16.2
Statin therapy	5.3	3.0–9.1
Diabetes	6.4	3.3–14.7
Hypertension	3.9	1.0–7.1
Smoking	3.2	1.9–6.3
Severe renal insufficiency	4.1	2.1–9.2

**Table 4.** **Multivariate analysis, odds ratio for a calcium score above 400 for different risk factors after adjustment for age, sex hyperlipidemia, diabetes, hypertension, smoking, severe renal insufficiency**.

	Odds ratio	*p*-value
Age	1.2 [1.02–1.18]	<0.001
Male sex	1.4 [1.08–1.39]	<0.001
Hyperlipidemia	3.8 [2.41–4.86]	<0.001
Statin therapy	2.8 [2.07–3.51]	<0.001
Diabetes	3.5 [2.25–4.56]	<0.0001
Hypertension	2.2 [1.65–3.08]	<0.001
Smoking	2.4 [1.78–3.30]	<0.001
Severe renal insufficiency	2.6 [1.91–3.27]	<0.001

Table 5 shows the hazard ratio for cardiovascular events in different score groups in group 1 and group 2. The hazard ratio was adjusted for sex, age, and coronary risk factors including hypertension, hypercholesterolemia, diabetes and smoking. In both groups we found a strong correlation between an increasing CAC score and an increasing hazard ratio for cardiovascular events. No cardiovascular events were observed in all patients without coronary calcification. The hazard ratio for cardiovascular events increased constantly with the CAC score in both groups, up to 7.3 (95% CI 4.0–10.8) in group 1 and 9.3 (95% CI 3.6–12.4) in group 2 for patients with a score above 1000. In corresponding score groups, the hazard ratio in patients with regular to moderately reduced renal function, group 2, was significantly higher compared to patients with severely impaired renal function.

**Table 5.** **Cox proportional hazards model predicting cardiovascular events in different calcium score groups for patients with normal to moderate reduced renal function and severe chronic kidney disease**.

Calciumscore	Severe CKD				Normal to moderately reduced renal function				
	Patients (n)	Events (n)	Hazard ratio	95% CI	Patients (n)	Events (n)	Hazard ratio	95% CI	*p*-value
0	44	0	1		136	0	1		
1–100	60	7	1.1	0.7–2.9	250	5	1.3	0.5–2.9	0.05
101–400	123	14	2.2	0.7–3.3	154	31	4.3	2.1–7.3	0.01
400–1000	95	32	4.5	2.1–8.4	91	34	6.8	3.8–10.2	0.01
>1000	71	28	7.3	4.0–10.8	70	32	9.3	3.6–12.4	0.01

CKD, chronic kidney disease.

## 4. Discussion

CAC is a well-established risk marker for future cardiovascular events. In previous studies, CAC demonstrated a more accurate and individual risk stratification for future cardiovascular events compared to score systems using cardiovascular risk factors such as the ATP III risk score or PROCAM score [3, 4, 5, 14].

The aim of this study was to evaluate whether CAC is a reliable marker in patients with severe CKD. Our study population consisted of asymptomatic patients who had no signs of prior coronary artery disease.

Cardiovascular risk factors were similarly distributed in both groups. There was a significantly higher number of patients on statin therapy in the group with severe CKD with a similar distribution of hyperlipidemia. This may be due to more frequent blood checks in the group of patients with severe CKD, which then lead to tighter control of lipid levels and more frequent administration of statins.

Severe CKD was associated with increased CAC compared to patients with normal to moderately reduced renal function. In patients with severe CKD, we could demonstrate that conventional cardiovascular risk factors led to higher amounts of CAC (Table 3). In addition, elevated CAC was found with increasing age and in male patients. These findings are consistent with previous studies in patients without severe CKD [4, 15]. Severe CKD itself could be identified as an independent risk factor for coronary calcification and as an independent risk factor for cardiovascular events.

Due to the protective effects of gestagen/progesteron on atherosclerosis, the CAC of females is lower compared to males in the same age group [16]. We found a higher calcium score in males, both in the normal to moderately reduced renal function group and in the severe CKD group. Despite a higher overall score, the difference was smaller in patients with severe CKD. This shows that the protective effect of the female sex is less relevant in this group. Due to the additional calcification induced by renal insufficiency, which occur regardless of gender, the scores could be slowly adjusted.

As demonstrated in prior studies [15, 17], patients with a CAC score of zero had a very low risk for future cardiovascular events (Table 5). Patients with a calcium score of zero on two consecutive examinations showed the best coronary disease prognosis [17]. CT angiographic studies have shown that patients with a score of 0 can have non-calcified plaques, but usually without relevant stenosis. Overall, however, the calcium score correlates with the extent of total atherosclerosis, so that a score of 0 indicates a low risk of atherosclerosis.

Over a follow up of 6.2 years in both groups, no patient with a CAC score of zero suffered from a cardiac event in our study. This demonstrates the excellent negative predictive value of a CAC score of zero even in high-risk populations. All 44 patients with a CAC score of 0 in the severe CKD group had no cardiovascular events during follow up regardless of existing risk factors.

Calcification is an almost ubiquitous pathological process in patients with end stage renal disease [9]. Therefore, the higher amount of CAC in the severe CKD group is not surprising. In severe CKD patients, the cardiovascular risk increased with an increasing CAC score (Table 5). Still, the hazard ratio of cardiovascular events in corresponding groups was lower than in patients with normal to moderately reduced renal function. In these patients, the equivalent CAC score was associated with a higher risk compared to patients with severe impaired renal function (Table 5). This could be derived from severe CKD specific calcifications in the media (Mönckenberg’ sclerosis), that might not contribute to additional cardiovascular risk at a similar level of conventional atherosclerosis.

It is known that the incidence of major cardiovascular events increases with higher CAC scores [18]. Therefore, in patients with severe CKD, the CAC score slightly overestimates the future cardiovascular risk compared to patients with normal to moderately reduced renal function because specific calcifications in the media are included in the CAC. In these patients with severe CKD, the CAC score is a valuable tool for cardiovascular risk assessment and the risk stratification for future events, especially with its high negative predictive value in patients with a score of 0. As described in other populations, in patients with severe CKD, the risk for cardiovascular events increased consistently with CAC. Therefore, it can be used to guide risk reduction therapy, for example lipid lowering with statin therapy. It is reasonable to assume that the number needed to treat to prevent cardiovascular event decreases with higher CAC scores, as shown in a previous study [19].

## 5. Limitations

Firstly, this is a single center study from Germany consisting of patients sent to our institution for a cardiological examination. Therefore, this cannot be considered an unselected population. Still, it can be regarded as a homogenous population without signs of CAD on study entry, as ECG, stress ECG, and echocardiography showed normal findings.

Secondly, due to the concentration of patients with CKD, the number of patients in this study is relatively small compared to other studies on coronary artery calcifications and cardiovascular events. However, the distribution of conventional risk factors is consistent with a typical population with cardiovascular risks in Europe. All patients received treatment according to current guidelines.

Thirdly, cardiac death was confirmed by autopsy in only 27% of patients, nevertheless in 66%, a prior MI was proven by ECG and laboratory testing. In 7%, clinical symptoms indicated a fatal infarction. So, it is reasonable to assume a cardiac death occurred in these patients.

## 6. Conclusions

In our asymptomatic high-risk population, we could demonstrate the importance of values for future cardiovascular events. A CAC of 0 in this high-risk group is associated with an excellent prognosis for event free progression. We could also demonstrate that risk prediction with CAC is still possible and independent in patients with severe CKD even if the CAC in patients with severe renal dysfunctions overestimated the risk for future cardiovascular events in comparison to patients with normal to moderately reduced renal function.

## Availability of Data and Materials

The datasets used and/or analyzed during the current study are available from the corresponding author on reasonable request.
